# *In vitro* activity of curcumin in combination with epigallocatechin gallate (EGCG) versus multidrug-resistant *Acinetobacter baumannii*

**DOI:** 10.1186/1471-2180-14-172

**Published:** 2014-06-27

**Authors:** Jonathan W Betts, David W Wareham

**Affiliations:** 1 Antimicrobial Research Group, Centre for Immunology and Infectious Disease, Blizard Institute, Queen Mary University London, 4, Newark Street, Whitechapel, London E1 2AT, UK; 2 Division of Infection, Barts Health NHS Trust, London E1 2ES, UK

**Keywords:** Curcumin, Epigallocatechin gallate, *Acinetobacter baumannii*, Synergy, Antibacterial

## Abstract

**Background:**

*Acinetobacter baumannii* is an opportunistic human pathogen often associated with life-threatening infections in the immunocompromised and the critically ill. Strains are often multidrug-resistant (MDR) and due to the lack of new synthetic antimicrobials in development for treatment, attention is increasingly focused on natural compounds either as stand-alone or adjunctive agents. Curcumin (CCM) is a natural polyphenol found in turmeric and isolated from the plant, *Curcuma longa*. Curcumin has been found to possess many biological properties, including antibacterial activity. In this study the antimicrobial activity of CCM and synergistic effects with epigallocatechin gallate (EGCG) against multidrug-resistant strains of *A. baumannii* were investigated and assessed via checkerboard and time-kill assays.

**Results:**

The MIC of CCM was >256 μg/mL against all strains of *A. baumannii* whilst those for EGCG ranged from 128-1024 μg/mL. In checkerboard studies synergy was observed against 5/9 isolates, with an additive effect noted in the remaining 4. The addition of EGCG reduced the MIC of CCM by 3- to 7-fold, with the greatest interaction resulting in a CCM MIC of 4 μg/mL. Time-kill curves indicated that a CCM-EGCG (1:8 and 1:4) combination was bactericidal with a 4 to 5-log reduction in viable counts after 24 h compared to the most effective polyphenol alone.

**Conclusions:**

This study demonstrates that despite little antibacterial activity alone, CCM activity is greatly enhanced in the presence of EGCG resulting in antibacterial activity against MDR *A. baumannii*. The combination may have a potential use in medicine as a topical agent to prevent or treat *A. baumannii* infections.

## Background

*Acinetobacter baumannii* is a non-fermentative Gram-negative bacterium that has emerged as a troublesome opportunistic human pathogen associated with life-threatening infections in the immunocompromised and critically ill [1]. It is a cause of bloodstream, respiratory, surgical and burn wound infections, often associated with medical devices. In recent years multi-drug resistant (MDR) strains have disseminated worldwide [2]. *A. baumannii* is intrinsically resistant to many antimicrobial compounds but also has a remarkable capacity to capture and sustain antimicrobial resistance determinants [2]. MDR strains are able to evade the effects of most antibiotics through a combination of enzymatic inactivation (β-lactamases, aminoglycoside modifying enzymes), impermeability (porin loss), chromosomal mutations and active efflux of drugs.

Due to the lack of new synthetic antimicrobials in development for the treatment of MDR Gram-negative infections, attention is increasingly focused on natural compounds either as stand-alone or adjunctive therapies. These include plant polyphenols such as those found in tea e.g. catechins and spices e.g. curcumin. Curcumin (CCM) is a diphenolic compound, commonly used in the form of turmeric throughout central and Eastern Asia as a spice and/or colouring agent in foodstuffs and textiles. A number of potential health benefits have been associated with CCM including anti-neoplastic, anti-inflammatory and anti-oxidant effects [3]. Studies have also revealed that CCM may have antimicrobial activity against both Gram-positive (*Streptococcus mutans*) [4] and Gram-negative bacteria (*Helicobacter pylori*) [5].

The antibacterial effects of CCM have also been shown to be affected when combined with other antimicrobials. Synergy has been observed when combined with oxacillin and ampicillin against meticillin-resistant *Staphylococcus aureus*[6] but antagonism when used with ciprofloxacin against *Salmonella typhi*[7].

Epigallocatechin-3-gallate (EGCG) is a polyphenol found in green tea, which like CCM, has been linked with health benefits and has significant antimicrobial activity against some MDR pathogens [8,9]. Previous studies have also shown that *A. baumannii* is inhibited by EGCG at concentrations between 78-625 μg/mL [10] and that the compound may act as an inhibitor of chromosomal penicillinase in *S. aureus*[11].

The potential for polyphenols to be used together against MDR Gram-negative bacteria was demonstrated previously, whereby potent synergy was observed when epicatechin was combined with theaflavin against *A. baumannii* and *Stenotrophomonas maltophilia*[12].

The bioavailability of natural compounds such as polyphenols and curcumin has been previously investigated and found to be in some cases their ‘Achilles heel’. Several studies have reported that although polyphenols penetrate effectively into various tissues [13] their bioavailability is poor [14] and it is difficult to achieve adequate concentrations for antimicrobial activity in mammalian models [15]. This may be a facet of their ability to bind to proteins [16] although many polyphenols are also rapidly metabolised in mammals [17]. If polyphenols are not absorbed before the small intestine, they are readily hydrolysed to simple phenolics by bacteria in the human microflora [18] further reducing their systemic bioavailability. Although phase 1 clinical trials have found that high doses (12 g/day) of systemic CCM are safe [19], the use of polyphenols as antimicrobials is likely to be limited to use as topical agents. The toxicity of EGCG was limited to minor skin irritation in mammalian models [20] at high concentrations and no adverse effects were seen with preparations containing up to 500 mg/Kg/day.

In this study we present data on the activity of CCM alone and in combination with EGCG against a well characterised collection of MDR *A. baumannii* clinical isolates.

## Methods

### Chemicals reagents and media

Curcumin powder (≥90% purity) extracted from *Curcuma longa* was purchased from the Cayman Chemical Company (Michigan, USA). Epigallocatechin gallate (≥95% purity) was donated by Unilever PLC (Bedford, UK). All growth media (Iso-Sensitest broth) was purchased from Thermo Scientific (Basingstoke, UK), sterilised and made up locally according to the manufacturer’s instructions.

### Bacterial strains

Nine *Acinetobacter baumannii* isolates were studied. These included the antibiotic susceptible type strain ATCC 19606 and 8 MDR clinical isolates. These have been extensively characterised previously and were chosen to be representative of UK epidemic clones (OXA-23 clones 1, 2, ‘Burn’) and/or exhibit resistance to colistin, tigecycline or produce metallo-β-lactamases (NDM enzymes) [21] Properties of the strains are detailed in Table 1. All isolates were stored at -70°C in microbank vials (Thermofisher, UK) and thawed prior to their use.

**Table 1:** **Resistant determinants and sources of multidrug-resistant clinical isolates of *Acinetobacter baumannii***

**Isolate**	**Properties**	**Isolate source**
AB 19606	Antibiotic Susceptible type Strain.	National Collection of type cultures
AB 14	MDR PFGE defined UK OXA-23 clone 1 OXA-23-like carbapenemase producer.	Dr J Turton, Public Health England, Colindale, UK
AB 16	MDR PFGE defined UK OXA-23 clone 2 OXA-23 carbapenemase producer.	Dr J Turton, Public Health England, Colindale, UK
AB 186	MDR PFEG defined UK ‘burn’ strain, OXA-23 producer.	Dr J Turton, Public Health England, Colindale, UK
AB 202	Tigecycline-resistant strain UK OXA-23 clone 1 isolate.	Barts Health NHS Trust, London, UK
AB 205	Colistin resistant UK OXA-23 clone 1 isolate.	Barts Health NHS Trust, London, UK
AB 292	MDR PFGE-defined OXA-23-like carbapenemase producer.	Barts Health NHS Trust, London, UK
AB 306	MDR NDM-1 carbapenemase producer.	Barts Health NHS Trust, London, UK
AB 308	MDR NDM-2 carbapenemase producer.	S. Gottig, Goethe Universistat, Frankfurt, Germany

### Determination of minimum inhibitory concentrations

Minimum inhibitory concentrations (MICs) were determined in corning 96-well microtitre plates (Corning, Amsterdam, The Netherlands). MICs of EGCG, CCM and combinations of both polyphenols were determined against all nine isolates according to British Society of Antimicrobial Chemotherapy (BSAC) susceptibility testing guidelines [22]. Doubling dilutions of CCM and EGCG stock solutions were added to horizontal wells in individual microtitre plates resulting in final concentrations ranging from 256-0.5 μg/mL (CCM) and 1024-2 μg/mL (EGCG). Equal volumes of *A. baumannii* (10^5^ CFU) in Iso-Sensitest broth were added to each well. After incubation at 37°C for 24 h in air, wells were checked for turbidity and the MIC recorded as the lowest concentration where no bacterial growth was observed. All microtitre assays were performed in triplicate and mean values presented.

### Determination of in vitro synergy of CCM-EGCG combinations

Synergy between CCM and EGCG was assessed in checkerboard assays, with doubling concentrations of CCM in vertical wells (256-4 μg/mL) and EGCG in horizontal wells (1024-1 μg/mL). Wells were inoculated with 10^5^ CFU of each *A. baumannii* isolate, incubated and analysed for growth as above. All assays were repeated in triplicate. Where the MIC was not reached, the concentration 1 dilution above the highest tested was used in assessing the strength of antimicrobial interactions.

Synergy between CCM and EGCG was determined by calculation of the Fractional Inhibitory Concentration Index (FICI) as previously described [1] whereby:

$$\begin{array}{l} \mathrm{FICI}=\frac{\mathrm{MIC}\;\mathrm{of}\;\mathrm{Compound}\;A\;\mathrm{in}\;\mathrm{combination}}{\mathrm{MIC}\;\mathrm{of}\;\mathrm{Compound}\;A\;\mathrm{alone}}\\ \;+\frac{\mathrm{MIC}\;\mathrm{of}\;\mathrm{Compound}\;B\;\mathrm{in}\;\mathrm{combination}}{\mathrm{MIC}\;\mathrm{of}\;\mathrm{Compound}\;B\;\mathrm{alone}} \end{array}$$

Synergy between the two compounds was defined as a FICI of ≤ 0.5, > 0.5-1.0 as an additive effect, > 1.0-4 as an intermediate effect and a value of > 4 suggestive of antagonism between the two compounds [23].

### Time-kill assays

Time-kill assays were undertaken using the antibiotic susceptible type strain (AB19606) and MDR isolate AB292 to determine the bactericidal activity of CCM, EGCG and a CCM-EGCG combination. Isolate AB292 was selected for use in time-kill as it is harbours a common MDR resistance profile, belongs to an epidemic clone (UK OXA-23 clone 1), but had similar MICs for CCM and EGCG as *A. baumannii* ATCC 19606. A 1 in 1000 dilution of an overnight culture of AB19606 and AB292 in Iso-Sensitest broth (10^6^ CFU/mL) was performed before the addition of CCM (0.25 × MIC), CCM (0.5 × MIC), EGCG (0.5 × MIC), EGCG (1 × MIC) or a combination of CCM-EGCG in a 1:4 ratio (w/w) and 1:8 ratio (w/w). Cultures (10 mL in universal bottles) were incubated at 37°C under continuous agitation for 24 h. At time intervals of 0, 2, 4, 6 and 24 h post inoculation, samples (100 μl) were collected, serially diluted and plated onto Iso-Sensitest agar. All inoculated plates were incubated at 37°C for 20 h before colonies were counted. Time-kill curves (CFU/mL v time) were plotted using GraphPad software. A difference of > 2 log_10_ CFU/mL between the single polyphenol and the polyphenols in combination at 24 h was used to determine synergy [24].

## Results and discussion

The MICs of CCM and EGCG alone and in combination are shown in Table 2. CCM had little antibacterial activity against any of the *A. baumannii* isolates even at a concentration of 256 μg/mL. Due to the solubility of CCM in water based media, concentrations >256 μg/mL could not be tested. When calculating the FICI a CCM MIC one dilution above the maximum concentration tested was used (Table 2). MICs of EGCG ranged from 128–1024 μg/mL. The antimicrobial activity of CCM was much lower against *A. baumannii* than those reported for *S. aureus* (MIC = 125-250 μg/mL) [6] and *H. pylori* (5-50 μg/mL) [5]. This could reflect variations in the growth media, differences in lipopolysaccharide (LPS) or cell wall architecture as well as penetration and transport of CCM across the Gram-negative outer membrane, issues well known to mediate resistance in *A. baumannii*[25].

**Table 2:** **Minimum inhibitory concentrations (MICs) of curcumin, epigallocatechin gallate and combinations of both compounds and fractional inhibitory concentration indexes (FICIs) versus *Acinetobacter baumannii***

**Isolate**	**MICs in monotherapy (μg/mL)**		**MICs in combination (μg/mL)**		**FICIs**
	**CCM**	**EGCG**	**CCM**	**EGCG**	
AB 19606	>256	1024	4	256	0.258 (S)
AB 14	>256	1024	4	512	0.508 (Ad)
AB 16	>256	1024	32	512	0.56 (Ad)
AB 186	>256	512	64	128	0.38 (S)
AB 202	>256	1024	64	512	0.63 (Ad)
AB 205	>256	1024	4	512	0.508 (Ad)
AB 292	>256	1024	4	256	0.258 (S)
AB 306	>256	128	4	32	0.258 (S)
AB 308	>256	256	4	64	0.258 (S)

MICs were within +/-1 dilution on replicate tests. CCM = curcumin, EGCG = epigallocatechin gallate, S = synergy, Ad = additive effect.

Several mechanisms for the antibacterial activity of CCM have been proposed including disruption of core metabolic pathways involved in folic acid metabolism (shikimate dehydrogenase) [5] and bacterial cell division (FtsZ) [26].The MICs of EGCG against the *A. baumannii* isolates used in our study were also higher than those previously reported [10] although it should be noted that the isolates tested in our study belonged to extensively resistant clones.

In combination tests, increased antibacterial activity was observed, with MICs for the combination being significantly lower than those for individual compounds. The addition of EGCG reduced the MIC of CCM by up to 3 -7 fold and was as low as 4 μg/mL for several isolates. Synergy between the two polyphenols was observed against five isolates (FICI ≤ 0.5) including one of the OXA-23 clone 1 isolates and the two NDM producers. An additive effect was observed with the remaining 4 isolates (Table 2). These results indicate that combinations of CCM and EGCG synergistically inhibit the growth of *A. baumannii* and that no antagonism occurs. This adds to previous research which showed synergy between natural compounds including tea polyphenols [12], where the addition of epicatechin, a compound with no antimicrobial activity against *A. baumannii* potentiated the activity of theaflavin.

The FICI as a measure of synergistic activity has limitations and more conservative limits of interpretation have been suggested [27]. The susceptibility breakpoint index (SBPI) may be a more useful parameter to assess positive interactions and the clinical usefulness of antimicrobial combinations [28]. However, as there are no breakpoints for EGCG and CCM susceptibility for *A. baumannii* or any other bacterial species, it was not possible to calculate an SBPI in this study. Therefore time-kill assays were used as a more robust method of assessing synergy.

**Figure 1:**
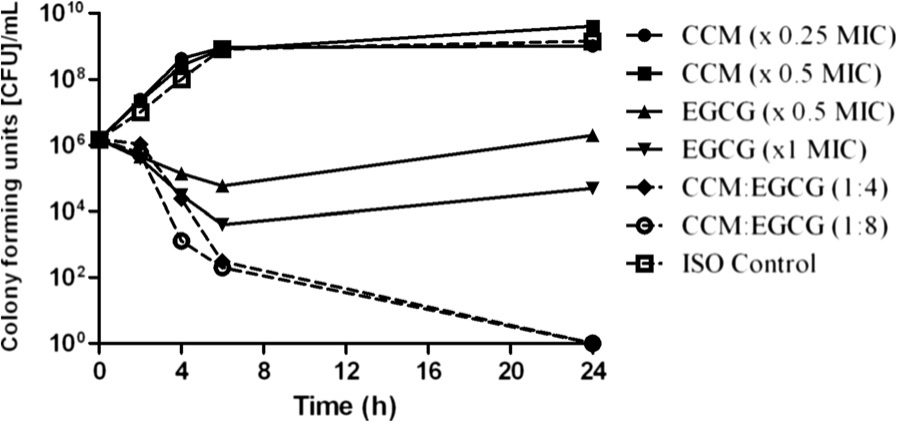
**Time-kill curve of *Acinetobacter baumannii *** **(ATCC 19606) versus CCM, EGCG and combinations of both compounds.**

**Figure 2:**
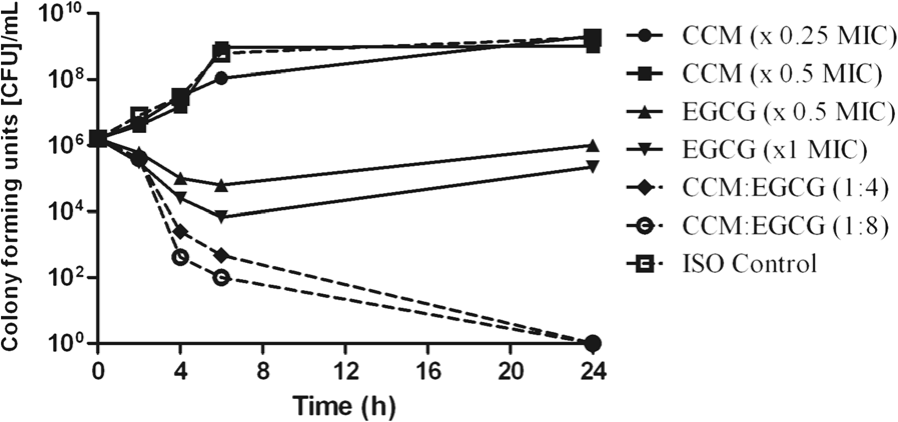
**Time-kill curve of *Acinetobacter baumannii *** **(AB292) versus of CCM, EGCG and combinations of both compounds.**

In time time-kill assays with the compounds used in 1:4 and 1:8 w/w (CCM:EGCG) ratios versus ATCC 19606 and AB292, a 4-5 log_10_ CFU/mL decrease was observed with the combination compared to the most effective polyphenol alone (Figure 1 and Figure 2) at 24 h. The combination had a sustained bactericidal effect up to and beyond 24 h post exposure whilst EGCG alone was only bacteriostatic, with regrowth observed after 6 hours exposure.

Although the mechanism for the antimicrobial synergy between CCM and EGCG has not been determined, it may involve disruption of the Gram-negative outer membrane combined with inhibition of essential proteins. Polyphenols including EGCG have a low affinity to bind LPS [29] but are able to act as pro-oxidants in the presence of metal ions. This may lead to increased H_2_O_2_ production and the formation of a hydroxyl radical, a mechanism shown previously to promote apoptosis in eukaryotic tumour cells [30] and outer membrane disruption/lysis of *Klebsiella pneumoniae and Escherichia coli*[31]. A possible explanation for the synergy between CCM and EGCG could therefore be disruption of the outer membrane via EGCG-led formation of H_2_O_2_ facilitating the entry of CCM into the cell. There is also evidence that antioxidants may protect each other from degradation [32,33] but further studies are required to investigate whether this phenomenon contributes to the enhanced antimicrobial activity of CCM in combination with EGCG”.

Although both EGCG and CCM-EGCG combinations have antimicrobial properties against MDR *A. baumannii,* both compounds have poor bioavailability. Due to this and the current solubility issues of CCM, any use would be limited to topical treatments. Although alone MICs are high, their clinical use as topical agents may still be possible as very high concentrations can be achieved locally [34]. In combination the concentrations required for antibacterial activity *in-vitro* are significantly lower and may be more readily obtained. The combination could have potential for the treatment and prevention of traumatic or burn wound infections and also as a coating on medical devices, surgical dressings, antimicrobial clothing [35] or as preservatives in foods to prevent spoilage.

The poor solubility of CCM in water is a limitation in determining *in-vitro* activity and may underestimate its biological activity. The development of water-soluble derivatives will be important in maximising the potential of this natural compound and previous studies have tried to address this [36]. Water-soluble curcumins have been developed as potential anticancer therapies although more cost effective and efficient methods are still needed for the extraction and modification of CCM. Although synergy between antimicrobial agents is important, the effect of antimicrobial combinations on bacterial killing and their ability to reduce antimicrobial resistance is crucial. Future studies should look into the effects of CCM in combination with other topical antimicrobial agents to further assess their potential as adjuncts for the treatment of MDR bacterial infections.

## Conclusions

Our study has shown that a combination of CCM and EGCG has an enhanced antimicrobial activity against multidrug-resistant *Acinetobacter baumannii*. This research suggests that the combination could be developed as an effective topical antimicrobial agent for the treatment and control of MDR Gram-negative infections in health and medicine.

### Ethics statement

As this was an entirely *in-vitro* study using bacterial isolates ethical review is not required.

## Competing interests

Both authors declare no conflict of interest in the design and execution of this study. No external funding was available to undertake this work.

## Authors’ contributions

JB carried out the experimental procedures, JB and DW designed the study and contributed equally to the analysis and production of the final manuscript.
